# Morphological Optimization of the Active Layer from Film Formation Kinetics in Organic Solar Cells

**DOI:** 10.1021/acsami.5c25668

**Published:** 2026-02-25

**Authors:** Jinsheng Zhang, Peter Müller-Buschbaum

**Affiliations:** Chair for Functional Materials, Department of Physics, TUM School of Natural Sciences, 9184 Technical University of Munich, James-Franck-Straße 1, 85748 Garching, Germany

**Keywords:** organic solar cells, crystallization, phase separation, kinetics, morphology

## Abstract

A deep understanding of the structural evolution is critical for the precise morphological optimization of the active layer in organic solar cells. This perspective systematically reviews the kinetic mechanisms of film formation and emphasizes that regulating processing parameters, such as deposition temperature, processing solvent, and additives, can effectively govern crystallization and phase separation during layer deposition. Advanced *in situ* characterization techniques are highlighted as indispensable tools for real-time decoding of these coupled kinetic pathways. We also summarize these techniques, covering their principles, detectable structural information, and inherent limitations and constraints, thereby guiding the selection of appropriate *in situ* tools. Finally, we outline the key challenges in the field and provide insights into future research aimed at advancing device performance through targeted morphological control.

## Graphical abstract

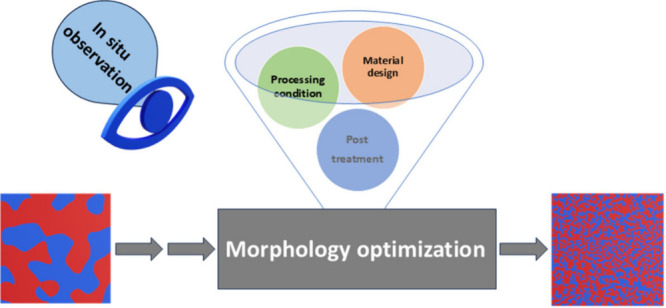

## 1 Introduction

Organic solar cells (OSCs) have garnered increasing interest due to their inherent advantages, including flexibility, solution processability, and continually improving power conversion efficiencies (PCEs). As the photon absorber, the active layer plays a critical role in converting solar energy into electrical energy. This active layer typically adopts a bulk heterojunction structure composed of two components: an electron donor and an electron acceptor. The donor–acceptor interfaces effectively overcome the high exciton binding energy and facilitate the dissociation. Given the limited exciton diffusion length, the phase-separation domains must be confined to an optimal nanoscale that simultaneously ensures continuous charge-transport pathways. Besides, the ordered molecular stacking can also provide faster charge mobility. Consequently, the morphological optimization of the active layer has emerged as the most common and effective strategy for enhancing OSC performance. A thorough understanding of the morphological evolution enables precise control over key structural features, such as crystallinity, molecular orientation, and domain size.

Active layer formation is an extremely complex kinetic process, which results from multiple coupled phase separations and crystallization. During the solvent evaporation, thermodynamic differences among the donor, acceptor, processing solvent, and additives significantly influence the domain size and purity. The separated phases subsequently form either amorphous aggregates or internal ordered structures that continue to grow over time through molecular diffusion and rearrangement. Some molecules with a planar structure even stack with each other in the early drying stage, which further competes with phase separation. To minimize surface free energy, smaller aggregates spontaneously dissolve and redeposit onto larger ones.^1^ Moreover, the interactions between materials also shape the final crystal orientation and stacking mode, including π–π and lamellar stacking. Understanding these kinetic events is essential for rational morphology control. *In situ* measurement techniques are indispensable tools for probing the morphological evolution in real time and elucidating the underlying film formation mechanisms. Unlike *ex situ* methods, *in situ* characterization demands a high response rate, low noise at weak signals, compatibility with wet film, etc. Figure 1 lists the commonly used *in situ* characterizations for OSCs. Among these methods, *in situ* UV–vis absorption spectroscopy is the most widely utilized to monitor the aggregation process due to its straightforward laboratory implementation. *In situ* light reflectometry enables thickness monitoring during drying, though refractive index variations from a wet to dry film may cause deviations between calculated and actual thickness evolution.^2,3^ *In situ* laser scattering detects refractive index contrast between phases, indicating the onset of phase separation.^4^ Since photoluminescence (PL) spectra reveal vibronic peak shifts associated with molecular aggregation, *in situ* PL can track aggregation behavior and molecule–environment interactions.^5^ Besides, *in situ* UV–vis absorption and *in situ* PL are more accessible in our standard laboratories; thus, synergistically combining them plays an important role in decoding film formation. For instance, a red-shifted absorption peak coupled with delayed PL quenching indicates that aggregation precedes donor–acceptor intermixing, revealing a two-stage process, where molecules first aggregate within their own phase before intermixing. On the contrary, rapid PL quenching with minimal absorption changes suggests molecular-level mixing from early stages. *In situ* grazing incidence wide-angle X-ray scattering (GIWAXS) and *in situ* grazing incidence small-angle X-ray scattering (GISAXS) represent more advanced techniques capable of providing more detailed and precise structural information evolution, although their application is constrained by the requirement for synchrotron X-ray sources. In addition, *in situ* light reflectometry, laser scattering, and UV–vis absorption primarily characterize bulk properties of the active layer. While *in situ* PL also probes the bulk structure, it exhibits high sensitivity to surface features, as surface defects can induce quenching. For *in situ* GIWAXS and GISAXS, the probing depth can be tailored to target either surface or bulk information by adjusting the X-ray incident angle.^6^ In practice, the comprehensive decoding of the drying process necessitates the integration of multiple complementary *in situ* measurements.

**Figure 1:**
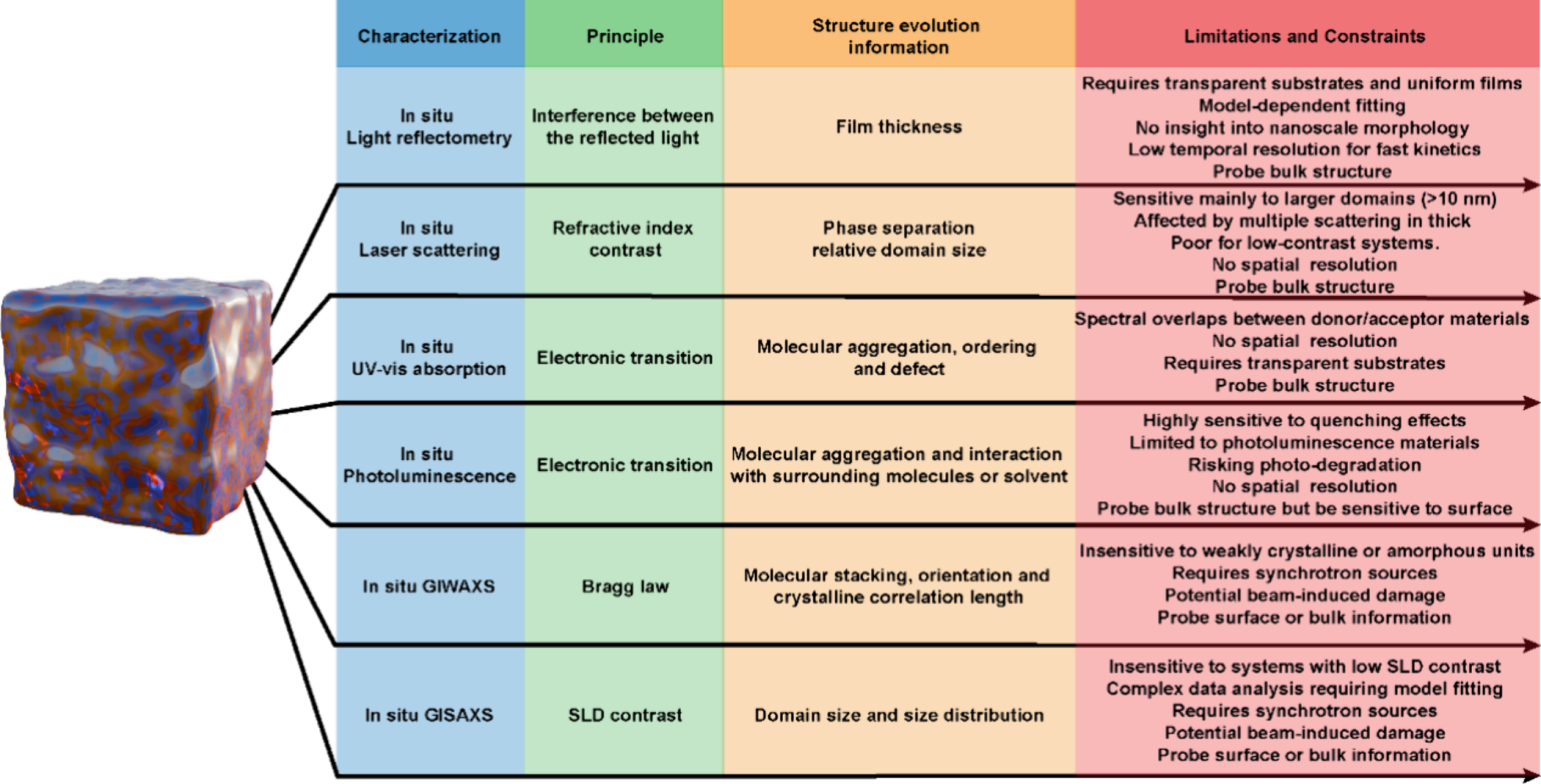
Summary of *in situ* measurement techniques, corresponding characterization principle, probed structural evolution information, limitations, and constraints.

Several factors can influence the film formation kinetics. First, the kinetic processes in fullerene-based systems differ distinctly from those in non-fullerene systems, resulting from the profound differences in their molecular properties. In fullerene systems, phase separation can be driven by polymer crystallization, a process that can squeeze fullerene aggregates out. In contrast, phase separation in non-fullerene systems is predominantly induced by spinodal decomposition. However, it is challenging to draw a specific distinction, as film formation kinetics are governed not only by the acceptor type. For a given donor/acceptor system, the kinetics can be deliberately tailored through processing parameters, most simply and commonly by varying the deposition temperature, solvent choice, and solvent additives. These parameters influence the drying period, crystallization sequences, and material miscibility, among others, thereby redirecting the pathway toward a refined final morphology. For instance, D18, recognized as a state-of-the-art donor material, has been extensively utilized to achieve excellent photovoltaic performance. The aggregation behavior of D18 plays a critical role in determining the final film morphology, which can be precisely modulated through various strategies, including solution heating, solvent additives, and a ternary blending strategy.^7–9^ Indeed, by optimization of the processing conditions, PCE exceeding 19% can be achieved even with non-BTP series acceptors. Besides, for the same active layer system and processing conditions, the film formation kinetic processes remain consistent regardless of the final device PCE. In this perspective, we systematically clarify how these processing parameters govern film formation kinetics, with a particular focus on their impact on phase separation and crystallization behavior in donor/acceptor blends. Additionally, we discuss the current theoretical model, which is used to analyze the drying process. Finally, we further propose that current challenges include experimental monitoring, model fitting, and accurately describing these complex kinetic processes. We offer perspectives to guide future kinetic research for targeted morphological optimization and improved OSC performance.

## 2 Crystallization

The formation of organic semiconductor thin films generally involves the self-assembly of molecules via intermolecular interactions, leading to semicrystalline structures. In OSCs, the molecular packing is characterized by π–π stacking and lamellar stacking, which can be effectively probed by using the GIWAXS method. The crystallization in organic systems typically proceeds via two key stages: nucleation and crystal growth. Among the theoretical models describing this process, the classical nucleation theory (CNT) is the most widely applied. CNT describes the homogeneous formation of spherical solid nuclei with radius *r* within a liquid parent phase. The Gibbs free energy change (Δ*G*) associated with nucleation is expressed as:

1 $$\Delta G=\frac{4}{3}\pi r^{3}\Delta G_{v}+4\pi r^{2}\gamma$$

Here, the first term corresponds to the volume free energy change (Δ*G*_v_), which is negative for a favorable phase transformation, while the second term represents the positive interfacial energy (γ).

This competition between the volume and surface effects is illustrated in Figure 2a. For small nuclei, the high surface-to-volume ratio causes the surface energy to dominate, resulting in a positive Δ*G*. As nuclei grow larger, the volume term becomes dominant, resulting in a negative Δ*G* and enabling stable crystal growth. Based on CNT, numerous *in situ* studies have been conducted to investigate how processing parameters affect nucleation and growth dynamics.

**Figure 2:**
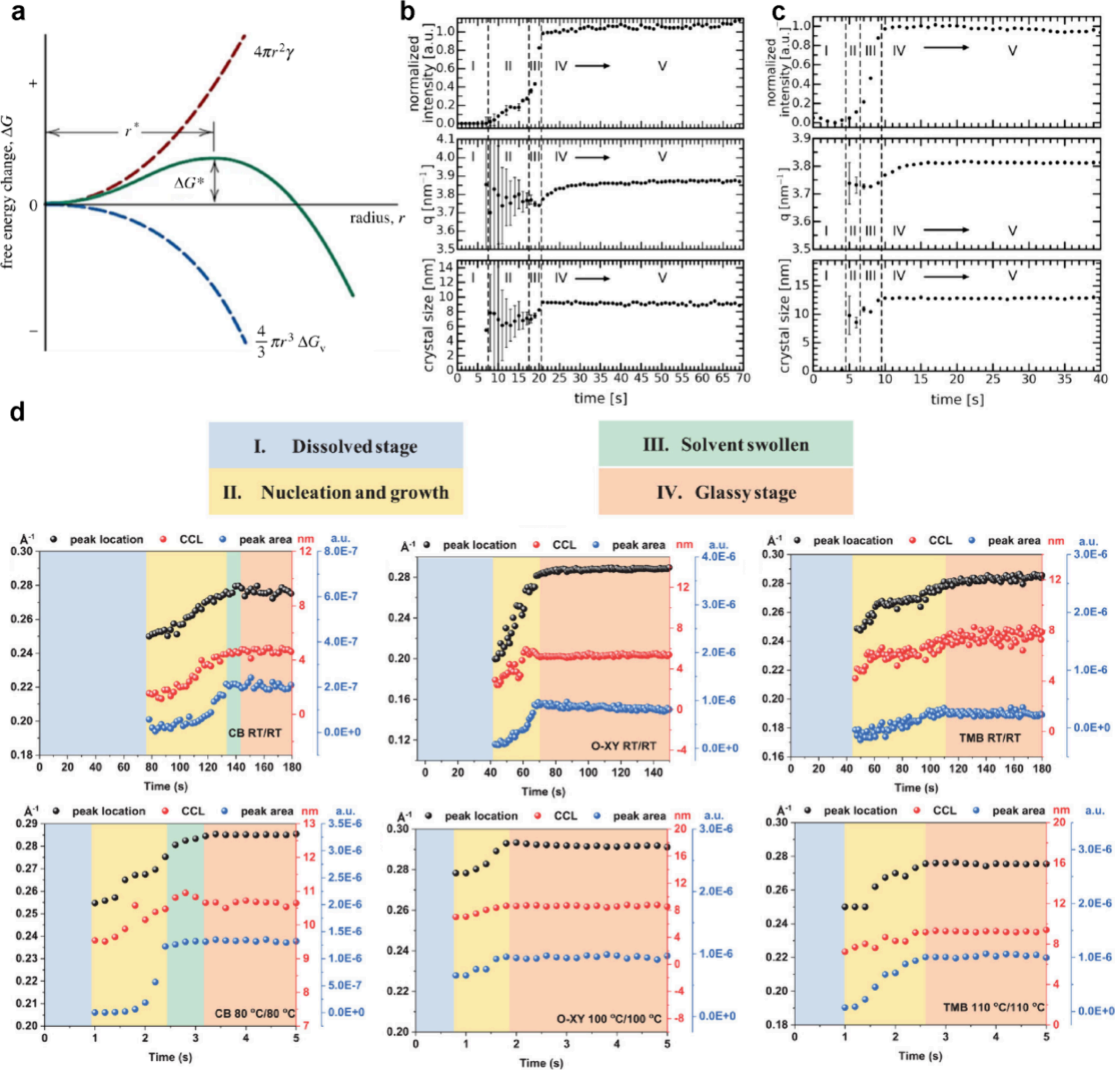
(a) Schematic plot of free energy versus nucleus radius. Panel a was reproduced with permission from ref 10. Copyright 2018 John Wiley & Sons, Inc. The temporal evolution of intensity, *q* value, and crystal size of the (100) peak extracted from *in situ* GIWAXS data at (b) room temperature and (c) 40 °C. Panels b and c were reproduced with permission from ref 11. Copyright 2015 Wiley-VCH Verlag GmbH & Co. KGaA. (d) Change of the peak position, CCL, and peak area over time at room temperature and elevated temperature in chlorobenzene, *o*-xylene, and 1,2,4-trimethylbenzene. Panel d was reproduced with permission from ref 12. Copyright 2020 Wiley-VCH GmbH.

Our group used *in situ* GIWAXS to investigate the crystallization of P3HT/PCBM from chlorobenzene and the related effects of the deposition temperature.^11^ Figure 2b and c shows the temporal evolution of the intensity, *q* value, and crystal size corresponding to the P3HT (100) peak. At room temperature, the drying process can be divided into five distinct stages. Stage I is a well-dissolved state. Crystallization begins at the end of this stage, when the solubility limit is reached. Influenced by the solvent scattering and low signals of the crystals, the initial crystals are generally ill-defined. Stage II can be attributed to nucleation, during which initial crystals exhibit suboptimal packing and a larger backbone spacing. Stage III involves rapid crystal growth, as evidenced by a sharp increase in scattering intensity and crystal size. In stage IV, solvent scattering disappears, crystal size and intensity saturate, and the (100) peak position gradually shifts due to polymer self-annealing. Finally, stage V corresponds to the completion of drying. When the substrate is heated to 40 °C, the same five-stage drying process is still observed but structural transformations are accelerated. Nucleation in stage II occurs much more rapidly than that at room temperature, leading to faster crystal formation and less optimal molecular packing, as indicated by increased stacking distances. However, higher temperatures increase the polymer chain mobility, leading to consistently larger crystal sizes throughout the crystallization process. Besides, in combination with *in situ* GISAXS data, it is found that fullerene molecules can be squeezed out of the polymer crystallization area and subsequently aggregate. Collaborative work with the Ma group reported similar phenomena, with detailed analysis of crystallization from three different solvents under both room-temperature and heating conditions.^12^ As shown in Figure 2d, the drying of PBDB-T-2F/BTP-4F from chlorobenzene follows a four-stage process, while the absence of a swollen state in *o*-xylene and 1,2,4-trimethylbenzene is attributed to the lower solubility of PBDB-T-2F (also designated as PM6) in these solvents. Elevated temperature deposition significantly shortens the drying time and increases the coherence lengths in all solvents. While crystallization patterns vary widely among solvents at room temperature, optimized temperature conditions lead to similar crystallization times (1 and 2 s) for chlorobenzene, *o*-xylene, and 1,2,4-trimethylbenzene, yielding films with comparable crystallinity. As a result, room-temperature-processed devices yield PCE values of 7.7, 5.3, and 6.3% for chlorobenzene, *o*-xylene, and 1,2,4-trimethylbenzene, respectively. After deposition temperature optimization, devices fabricated with all three solvents achieve both enhanced and similar performance, achieving PCE values of around 15.0%.

Beyond the processing temperature, solvent additives are commonly used to tune film morphology. According to their solubility and volatility, additives can be divided into four categories. Among them, additives with low volatility and selective solubility are the most effective at improving performance. The Liu group systematically investigated how 1,8-octanedithiol additives optimize the nanostructure formation of PBDB-T-2F/IT-4F films (Figure 3a).^13^ Treating the crystallization of PM6 during drying as an isothermal process, they analyzed the kinetics using the Johnson–Mehl–Avrami–Kolmogorov (JMAK) model

2 $$X\left(t\right)=1-\exp\left(-{kt}^{n}\right)$$

where *X*(*t*) is the transformed fraction, *k* is a rate constant dependent on nucleation and growth rates, and *n* is the Avrami exponent that provides information about the dimensionality of crystal growth (1D, 2D, or 3D) and the nature of the nucleation process (e.g., instantaneous, sporadic, or dependent on time). The transformation evolves through three distinct stages. It begins slowly and then accelerates as the expanding interfacial area provides more sites for atomic or molecular attachment. The process ultimately decelerates as the transformable material becomes scarce, and growing grains start to overlap with each other. Impingement manifests either through direct grain contact or remotely via overlapping diffusion fields, which causes grains to compete for available compositional resources. In practice, crystallization kinetics in OSC films are more complex due to multiphase interactions, which cannot be described by one single nucleation–growth model. When ln­[−ln­(1 – *X*(*t*))] vs In­(*t*) curves are plotted, distinct stages can be identified from the slope changes (Figure 2). Further decomposing the Avrami exponent indicates that stage I is nucleation-dominated and accelerates with 1,8-octanedithiol addition, while stage II is primarily interface-controlled crystal growth with negligible nucleation, showing less sensitivity to 1,8-octanedithiol. Our group also applied the JMAK model to study the effect of solvent additive selectivity on film formation, identifying five distinct stages based on slope variations seen in Figure 3b and c.^14^ Moreover, we used a multi-Gaussian peak fit to deconvolute π–π-stacking signals and applied batch-data processing to extract statistical trends. Results show that both studied additives, 1-chloronaphthalene and tetralin, significantly prolong the drying period of PBDB-T-2F/BTP-C3-4F in chloroform at room temperature. 1-Chloronaphthalene, which has a higher solubility for the acceptor, suppresses donor crystallization and promotes larger acceptor domains due to the additional space and time for molecular rearrangements provided by the residual additives. In contrast, tetralin, with inferior acceptor solubility, swells the polymer donor and breaks the acceptor crystals into smaller units. However, this significantly prolonged crystallization process leads to an unbalanced crystallinity between the donor and acceptor, resulting in highly distinct electron and hole transport that ultimately diminishes device performance. Appropriate thermal annealing can effectively remove the additives while enhancing the crystallinity without compromising balanced charge transport. Consequently, devices processed with both additives and thermal annealing achieve PCE values higher than those of the as-cast devices. Overall, several universal principles emerge from these crystallization studies. The film formation consistently follows a multistage progression, involving dissolution, nucleation, growth, and structural refinement, that can be effectively analyzed using the classical nucleation theory and the JMAK model. Processing parameters, such as the temperature and solvent additives, primarily influence crystallization through two mechanisms: controlling the crystallization rate and tuning the sequence of donor and acceptor ordering. Elevated temperatures accelerate crystallization but may compromise molecular packing, while selective additives can extend the crystallization window and promote component-specific ordering.

**Figure 3:**
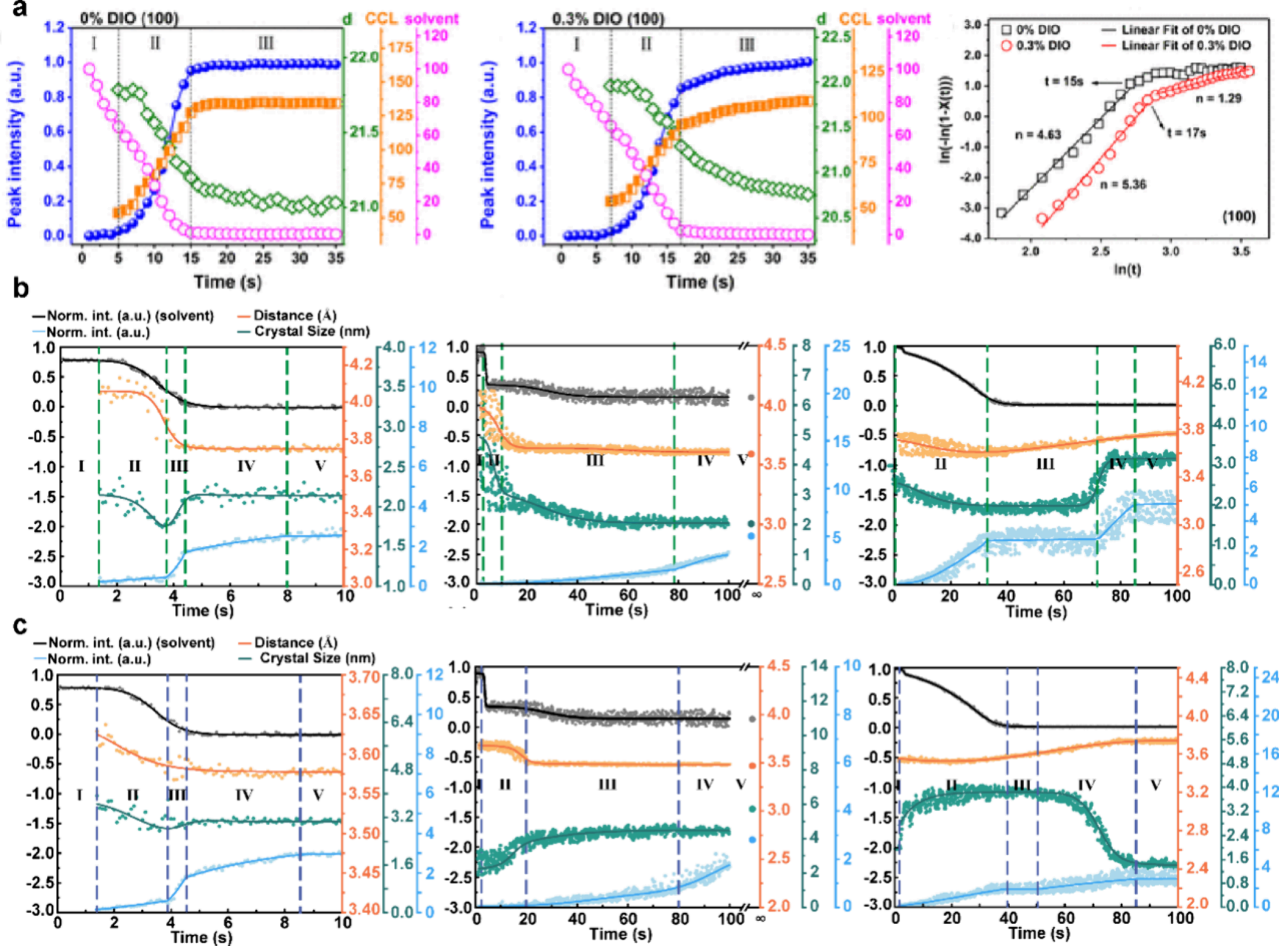
(a) Temporal evolution of the (100) peak in the PBDB-T-2F/IT-4F blend with and without 1,8-octanedithiol and the corresponding analysis results using the JMAK model. Panel a was reproduced with permission from ref 13. Copyright 2021 American Chemical Society. The π–π stacking evolution of the (b) donor and (c) acceptor during slot-die coating in chloroform with and without solvent additives at ambient conditions. Panels b and c were reproduced with permission from ref 14. Available under a CC-BY 4.0 license. Copyright 2024 Zhang et al.

## 3 Phase Separation

OSCs inherently are a two- or multiphase system, where phase separation inevitably occurs, driven by component immiscibility and differences in crystallinity. Optimizing the scale of phase separation is critical in OSCs. Excessively large phase separation hinders exciton diffusion to donor–acceptor interfaces, leading to recombination, while overly small domains impede efficient charge transport. As illustrated in Figure 4a, phase separation can proceed through liquid–liquid (L–L), liquid–solid (L–S), and solid–solid (S–S) pathways.^15^ L–L phase separation generally starts via spinodal decomposition or crystallization when the blend crosses from the one-phase to two-phase region. Spinodal decomposition is a barrier-free phase separation process in binary mixtures that occurs spontaneously when the reaction is quenched into the unstable spinodal region of the phase diagram. In contrast, the L–S separation occurs when the solubility limit of either component is exceeded during solvent drying. Even after L–L and L–S phase separations are complete, the morphology may remain metastable and continue to evolve through S–S separation via molecular rearrangement and mobility. For systems dominated by L–L phase separation, the evolution can be analyzed using a ternary phase diagram, governed primarily by interaction parameters (Figure 4b).^16^ The extent of L–L phase separation depends on the quench depth at which the blend enters the two-phase region during deposition. The quench depth refers to the degree to which a system is rapidly shifted from a thermodynamically stable to an unstable region of the phase diagram, typically via temperature, solvent evaporation, or concentration changes. Generally, highly compatible donor–acceptor pairs exhibit shallow quench depths, resulting in small phase separation. Additionally, rapid drying kinetics often lead to deep quench depths and large-scale phase separation.^17^ Conversely, in systems governed by L–S phase separation, prolonged drying allows more time for molecular aggregation and can also promote larger domains. Beyond the domain size, the domain purity has a significant impact on the OSC performance. In well-miscible systems, L–L phase separation tends to yield low-purity domains. Therefore, suppressing L–L separation while promoting L–S phase separation can be beneficial for achieving optimal morphology. However, in practice, L–S phase separation competes with L–L phase separation by depleting one solute from the liquid phase, thereby altering its composition and delaying L–L demixing. This competition adds more complexity to the overall film-formation kinetics.

**Figure 4:**
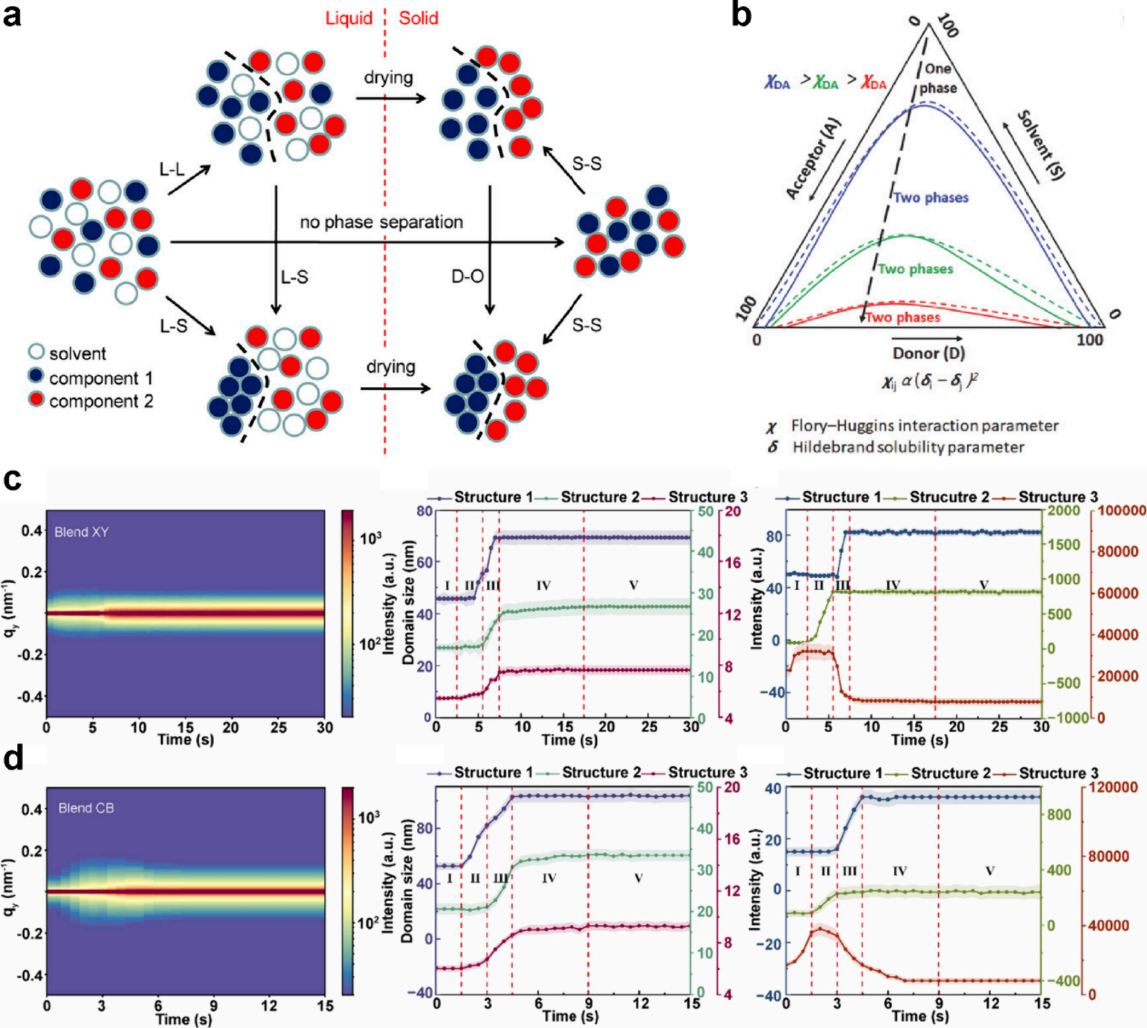
(a) Schematic of the solidification pathways through phase separation in a precursor solution containing two components. Panel a was reproduced with permission from ref 15. Copyright 2013 American Chemical Society. (b) Schematic ternary phase diagram for the OSC blend with varying donor–acceptor interaction parameters. Panel b was reproduced with permission from ref 16. Copyright 2018 Wiley-VCH Verlag GmbH & Co. KGaA. Temporal evolution of horizontal line cuts in *in situ* GISAXS and corresponding structural information in the case of (c) *o*-xylene and (d) chlorobenzene. Panels c and d were reproduced with permission from ref 18. Available under a CC-BY 4.0 license. Copyright 2024 Zhang et al.

Current binary non-fullerene OSCs typically consist of a polymer donor and a small-molecule acceptor, forming a polymer/solvent/non-solvent system. The phase separation through spinodal decomposition in such systems can be probed by using *in situ* GISAXS. Our group used this technique to successfully monitor the dynamic process of spinodal decomposition in a PBDB-TF-TTz/BTP-4F-24 system.^18^ Through deep model fits on the horizontal line cuts at the Yoneda peak, large, medium, and small structures, corresponding to structures 1, 2, and 3, respectively, can be extracted in detail. Their temporal evolution is shown in Figure 4c and d. With focus on the medium-length structure, its domain size remains constant in stage II, while its intensity increases exponentially. In stage III, the domain size grows sharply, while the intensity stays the same. These two stages align well with the early and late stages of spinodal decomposition, as described by the Cahn–Hilliard linearized theory and Furukawa dynamical scaling theory. In the early stage, concentration fluctuations grow exponentially in amplitude without a significant change in the domain size. In the late stage, domains undergo self-similar growth driven by interfacial energy minimization, leading to an increase in the domain size at a constant amplitude. The resulting worm-like morphology further confirms the spinodal decomposition mechanism. Besides, solvent selection also determines the different spinodal decomposition routes. The larger initial clusters in chlorobenzene can lead to stronger domain coarsening via Ostwald ripening, which finally causes larger phase separation compared to that in *o*-xylene. In addition, the Chen group used *in situ* UV–vis absorption spectroscopy to study the effect of drying time on the morphology of PBDB-T-2F/BTP-eC9 blends (Figure 5a).^19^ During the solidification process, the 0–0 vibrational transitions of the donor and acceptor undergo red shifts of varying extents, demonstrating the occurrence of phase separation. When low-boiling-point chloroform was used, film formation was completed within 4.7 s. With a higher boiling point, toluene allowed the process to be extended to 12.4 s, providing more time for molecular self-organization and yielding a highly crystalline morphology with smaller domains. Additionally, manipulating the phase-separation sequence provides a pathway to optimize the final film morphology. The Zhang group designed and synthesized a novel additive, 1,2-di­([2,3′bithiophen]-2′-yl)­ethyne (DBTE), which was introduced in the PBDB-T-2F/BTP-4F blend.^20^ *In situ* UV–vis absorption spectra (Figure 5b) demonstrate that the DBTE addition induces sequential aggregation, wherein BTP-4F aggregates after PBDB-T-2F, in contrast to the simultaneous co-aggregation observed in additive-free binary blends. This delayed acceptor aggregation facilitates continuous vertical migration, resulting in acceptor enrichment near the active layer surface. The resulting vertical phase separation simultaneously achieves high efficiency and photostability. Our group also applied *in situ* UV–vis spectroscopy to PBDB-T-2F/IT-4F blends.^21^ By deconvoluting the absorption spectra based on a simplified Franck–Condon principle (Figure 5c), we identified five distinct stages for both the donor and acceptor. Throughout film formation, the donor peak position remained constant, indicating small aggregates existing in the precursor solution. Moreover, the energy disorder of both components increased due to more complex interactions with the surrounding environment during solidification. Overall, phase separation pathways are governed by the competition among L–L, L–S, and S–S phase separation mechanisms, with the dominant route determined by material miscibility and processing conditions. *In situ* techniques, particularly GISAXS and UV–vis absorption, effectively capture these dynamics, revealing that processing parameters control phase separation through two key mechanisms: modulating quench depth and the sequence of donor versus acceptor aggregation. A consistent finding is that suppressing L–L demixing at an early stage while promoting L–S demixing enhances domain purity and device performance.

**Figure 5:**
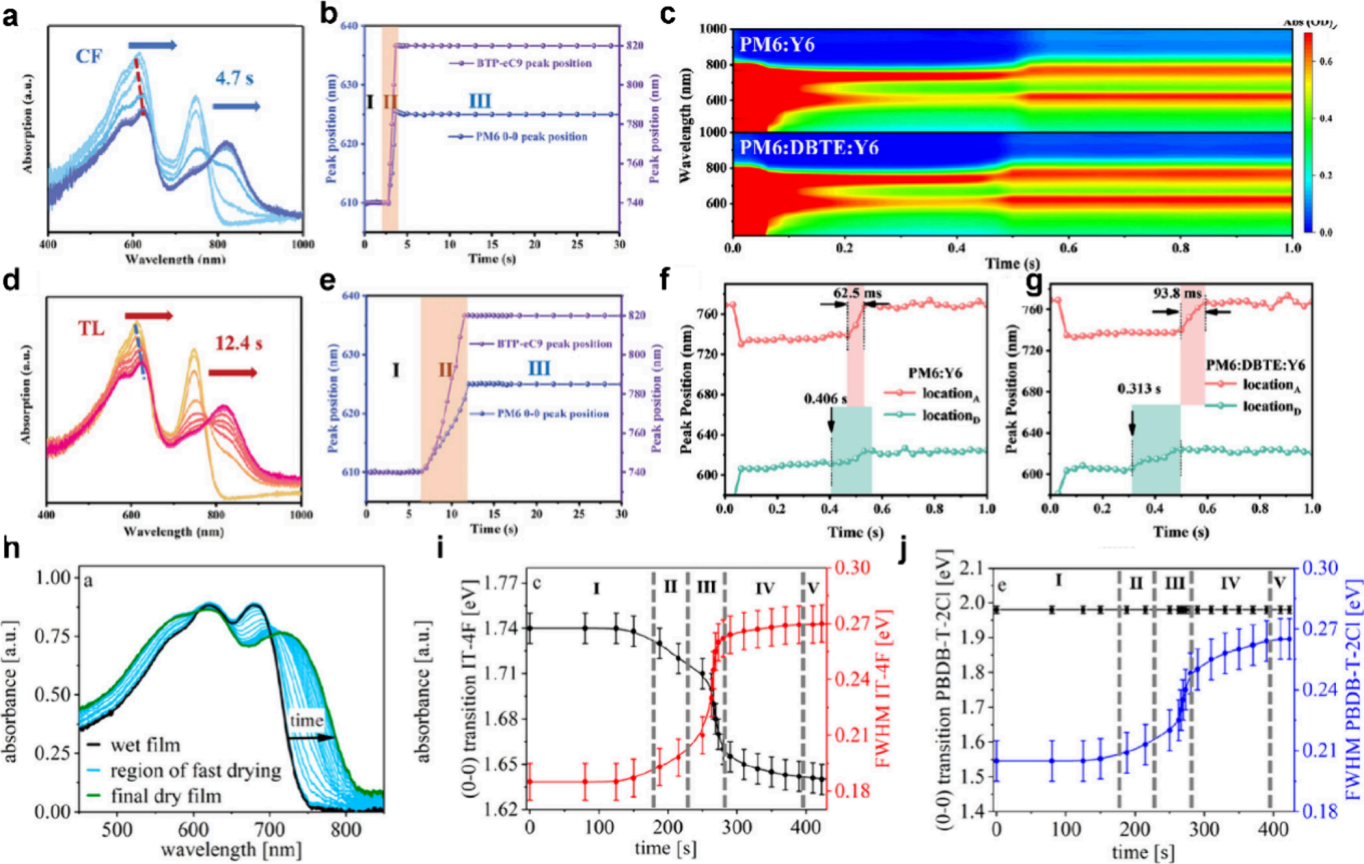
Temporal evolution of absorption spectrum for the PBDB-T-2F/BTP-eC9 blend in (a) chloroform and (d) toluene and the corresponding donor and acceptor peak position in (b) chloroform and (e) toluene. Panels a, b, d, and e were reproduced with permission from ref 19. Copyright 2022 Wiley-VCH GmbH. (c) Contour map of *in situ* absorption for the PBDB-T-2F/BTP-4F blend with and without additives. The evolution of the peak position in the PBDB-T-2F/BTP-4F blend (f) with and (g) without additives. Panels c, f, and g were reproduced with permission from ref 20. Copyright 2025 Wiley-VCH GmbH. (h) *In situ* absorption spectrum and corresponding evolution of PBDB-T-2Cl and IT-4F. The temporal evolution of the 0–0 transition for (i) IT-4F and (j) PBDB-T-2Cl. Panels h–j were reproduced with permission from ref 21. Copyright 2020 American Chemical Society.

## 4 Conclusion and Outlook

This perspective summarizes recent advances in understanding crystallization and phase separation kinetics during the film drying process with a particular emphasis on how *in situ* characterization techniques enable real-time monitoring of film formation dynamics for morphological optimization. Such *in situ* investigations significantly deepen our understanding of how processing parameters govern morphological evolution. Specifically, suitably elevating the deposition temperature can simultaneously enhance the crystal size and suppress excessive phase separation. Likewise, solvent selection critically influences the duration of phase separation and molecular interactions, thereby altering the solidification pathway. Solvent additives generally prolong the crystallization window and can selectively promote the growth of larger crystals in either the donor or acceptor component. Furthermore, current methods for identifying distinct stages rely heavily on subjective researcher interpretation. To address this challenge, we suggest a systematic three-step protocol for a more objective stage classification. First, it is essential to identify which structural parameters serve as the classification criteria. Film formation involves multiple structural characteristics, including the domain size, domain center-to-center distance, crystallinity, crystal size, molecular orientation, and so on. Since these parameters typically exhibit different evolution patterns, determining the main structural information as the classification basis is critical. Second, the evolution trends of selected structural parameters should be statistically analyzed to minimize random errors and ensure accuracy. This requires sufficient data points and an appropriate error analysis throughout the drying process. Third, suitable kinetic models should be applied to quantitatively analyze these statistical evolution trends. The insights gained from these processing–morphology correlations are also applicable to novel molecular design, such as optimizing the alkyl chain in state-of-the-art materials, such as BTP-4F. The chemical structures of photovoltaic materials fundamentally determine their molecular properties and ultimately influence film morphology. Common molecular design strategies include heteroatom substitution, conjugation length adjustment, and so on. For instance, rigid molecules are prone to more efficient intermolecular stacking and, thus, exhibit a greater propensity for crystallization. In addition, stronger intermolecular interactions between donor and acceptor components can lead to the formation of an overmixed morphology with poor domain purity. Establishing the relationship between these structural modifications and kinetic processes can guide rational material design and deepen our understanding of film formation mechanisms. However, most current studies rely on only one or two probing techniques to monitor the drying process, which imposes inherent limitations on their conclusions and often restricts their applicability to other material systems. In contrast, multiprobe studies that perform several *in situ* characterizations simultaneously can capture far richer information on the structural evolution, ranging from simple film thickness to the nanoscale structural evolution of organic semiconductors. These coupled results are beneficial for a deeper analysis of the interplay between crystallization and phase separation, enabling a more comprehensive and accurate depiction of the film formation kinetics. Additionally, devices with the highest PCE are currently produced by spin coating, a method limited to lab-scale fabrication. In contrast, printing technologies, such as blade coating and slot-die coating, enable large-scale device fabrication, making them more suitable for commercialization despite their currently lower PCE. Therefore, further research on printing kinetics to guide PCE improvement and facilitate industrial-scale production is essential. Besides, the layer-by-layer (LBL) heterojunctions are a structure intermediate between planar heterojunctions and bulk heterojunctions. It not only enables a certain degree of vertical phase separation to achieve excellent charge transport but also provides sufficient donor–acceptor interfaces to ensure efficient exciton dissociation. To date, remarkably high PCEs have been achieved based on this structure.^22^ However, *in situ* studies of its formation process remain relatively scarce. Therefore, exploring the film formation kinetics of LBL films is also of great significance. Furthermore, *in situ* experiments inherently generate vast amounts of data, complicating analysis. Developing batch-processing tools can greatly reduce repetitive and inefficient tasks. This is particularly crucial when spectral deconvolution or model fits are required, as manual processing of thousands of data points is often impractical. Appropriate tools can help preserve complete structural evolution information and enhance the reliability of the results. Beyond advancing current *in situ* setups, enriching the toolbox by developing existing *ex situ* methods into new *in situ* techniques will be key to uncovering the fundamental mechanisms of film formation. We believe that *in situ* characterization will continue to play an indispensable role in advancing the morphological understanding of OSCs and providing critical guidance for morphology optimization to enhance the performance and stability.
